# Comparison of Natural Killer Cells Differentiated from Various Pluripotent Stem Cells

**DOI:** 10.3390/ijms25158209

**Published:** 2024-07-27

**Authors:** Jongsuk Han, Hyeongbin Son, Daun Jung, Ki-Yeon Kim, Chaeyeon Jin, Hyeonwook Hwang, Soon-Suk Kang, Shoukhrat Mitalipov, Hee-Jung An, Yeonmi Lee, Eunju Kang

**Affiliations:** 1 Department of Biomedical Science, College of Life Science, CHA University, Seongnam-si 13488, Gyeonggi-do, Republic of Korea; flvm1023@gmail.com (J.H.); pos00248@naver.com (H.S.); cyjin1999@gmail.com (C.J.); gusdnr9528@naver.com (H.H.); 2 Department of Pathology, CHA Bundang Medical Center, CHA University, Sungnam-si 13496, Gyeonggi-do, Republic of Korea; jhd2800@chamc.co.kr (D.J.); superk8787@chamc.co.kr (K.-Y.K.); hjahn@cha.ac.kr (H.-J.A.); 3 Cell Therapy 3 Center, CHA Advanced Research Institute, CHA Bundang Medical Center, Sungnam-si 13488, Gyeonggi-do, Republic of Korea; soycorn@chamc.co.kr; 4 Center for Embryonic Cell and Gene Therapy, Oregon Health and Science University, Portland, OR 97239, USA; mitalipo@ohsu.edu; 5 Department of Biochemistry, School of Medicine, CHA University, Seongnam-si 13488, Gyeonggi-do, Republic of Korea

**Keywords:** pluripotent stem cell, natural killer cell, cancer immunotherapy, NK cell differentiation, B2M KO, phenotype analysis, cytotoxicity assay

## Abstract

Allogeneic natural killer (NK) cell therapy has been effective in treating cancer. Many studies have tested NK cell therapy using human pluripotent stem cells (hPSCs). However, the impacts of the origin of PSC-NK cells on competence are unclear. In this study, several types of hPSCs, including human-induced PSCs (hiPSCs) generated from CD34+, CD3−CD56+, and CD56− cells in umbilical cord blood (UCB), three lines of human embryonic stem cells (hESCs, ES-1. ES-2 and ES-3) and MHC I knockout (B2M-KO)-ESCs were used to differentiate into NK cells and their capacities were analyzed. All PSC types could differentiate into NK cells. Among the iPSC-derived NK cells (iPSC-NKs) and ESC-derived NK cells (ES-NKs), 34+ iPSCs and ES-3 had a higher growth rate and cytotoxicity, respectively, ES-3 also showed better efficacy than 34+ iPSCs. B2M-KO was similar to the wild type. These results suggest that the screening for differentiation of PSCs into NK cells prior to selecting the PSC lines for the development of NK cell immunotherapy is an essential process for universal allotransplantation, including the chimeric antigen receptor (CAR).

## 1. Introduction

Immune cell therapy (ICT) has emerged as a promising approach for treating cancer as well as combating pathogens and viruses. The clinical benefits of ICT have been demonstrated, with immune cells responding to disease in various ways within the host. This represents a significant advancement over traditional cancer treatments, such as surgery, chemotherapy, and radiation therapy [1]. With advances in immunology, genetic engineering, gene editing, and synthetic biology, ICT has emerged as a potential tool for cancer therapy. This is evident with the clinical success of chimeric antigen receptor (CAR)-modified T cells [2]. Numerous studies have reported positive effects in the clinical stages of FDA-approved CAR-T cell products, which are engineered from autologous T cells harvested from the patient. However, CAR-T cells have limitations, such as antigen escape, off-target effects, associated toxicities, and high production costs [3,4,5]. Due to these limitations, NK cells have recently emerged as an alternative.

NK cells are crucial components of the innate immune system, eliminating various tumors and viral infections through their cytotoxic functions [6]. Unlike T cells, NK cells can directly kill target cells without prior sensitization and have fewer side effects, such as cytokine syndrome and graft-versus-host disease [7,8,9], suggesting that NK cells could be a promising option for ICT. They have various receptors on their surface to identify target cells by recognizing specific ligands on those target cells [10]. Inhibitory receptors of NK cells, such as CD94-NKG2A and inhibitory members of killer Ig-like receptors (KIRs), including KIR2DL1 and KIR2DL2/3, bind major histocompatibility complex class I (MHC I) on target cells [11]. Activating receptors, such as NKp30, NKp44, and NKp46, as natural cytotoxicity receptors; NKG2D, NKG2C, and NKp80 as C-type lectin-like receptors; and DNAM1 and CD2 as coactivating receptors, detecting ligands on target cells and triggering cytotoxicity to eliminate them [12]. Through signaling balances between these inhibitory and activating receptors, NK cells decide to kill target cells [13,14,15,16]. Additionally, NK cells express activating Fcγ receptors, specifically FcγRIIIa, also known as CD16a. These receptors induce antibody-dependent cellular cytotoxicity when antibodies bind to antigens on target cells, thereby facilitating NK cell-mediated destruction of target cells [17,18].

PSCs are an effective source of off-the-shelf NK cells for allogeneic applications. The expression of functionally mature NK cell markers was similar between PSC-NK cells and peripheral blood (PB)-derived NK cells [19]. The NK cells derived from ESCs have been reported to be more effective than umbilical cord blood (UCB)-derived NK cells at killing K562 cells in a murine xenograft model [20]. Genetic modification was technically easier in the PSC state than in primary NK cells [21]. These advantages of PSC and NK cells are being used to conduct studies on genetically modified PSC-derived NK cells. Clinical studies using CAR-NK cells or hypo-immune PSC-NK cells are in progress [22,23,24].

MHC I, also known as HLA class I, is a crucial mediator of the immune response. It consists of three α domains and one β2-microglobulin (B2M). Disruption of the B2M gene leads to impaired MHC I assembly and subsequent suppression of MHC I expression. NK cells are considered reliable sources for allotransplantation because the mismatching of their inhibitory receptors with the recipient’s MHC could induce fewer immunological side effects in the recipient [25]. Furthermore, injected NK cells are also required to evade the recipient’s immune system. Disruption of MHC I in NK cells is thought to evade T cell-mediated rejection of the recipient [23,24,25]. Several studies have demonstrated that NK cells do not function properly when there is a lack of interaction between their inhibitory receptors and self-MHC I during maturation [26,27,28,29]. Therefore, it is important to determine whether the lack of MHC I affects the differentiation of PSCs into NK cells, and whether the differentiated cells function properly.

In this study, human PSCs derived from various origins were differentiated into NK cells. They used a protocol without feeder cells and embryoid body (EB) formation to compare their phenotypes, cell growth, and cytotoxicity. The feeder cells improve the cell growth of differentiated NK cells but make it difficult to separate from NK cells despite sufficient irradiation of feeder cells [30]. Additionally, this study evaluated whether B2M knockout (for depleting MHC I expression) could affect NK cell differentiation and function.

## 2. Results

### 2.1. Generation of iPSCs from UCB

**Figure 1:**
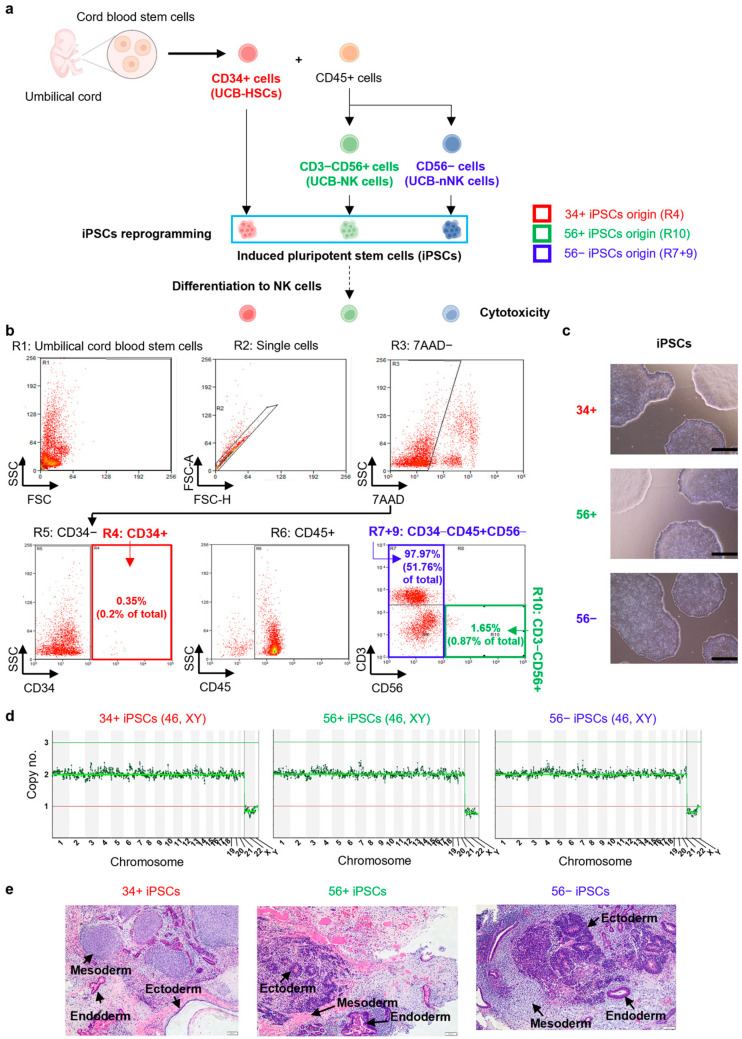
Isolation of cells from cord blood mononuclear cells and generation of iPSCs. (**a**) Schematic of the experiment. (**b**) The strategy of UCB cell sorting. (**c**) Morphology of iPSCs generated from 34+, 56+, and 56− of UCB MNCs. Scale bar: 50 μm. (**d**) Copy number variation profile of iPSCs. This indicates that all chromosomes are diploid. (**e**) Teratoma formation includes the three germ layers (ectoderm, mesoderm, and endoderm) from 34+ iPSCs, 56+ iPSCs, or 56− iPSCs. Scale bar: 200 μm.

To investigate the differentiation ability and function of PSC-NKs depending on the origin of UCB mononuclear cells (MNCs), the cells were separated into three types (CD34+ (34+), CD3−CD56+ (56+) and CD56− (56−)), generated iPSCs (34+ iPSCs, 56+ iPSCs, and 56− iPSCs), differentiated into NK cells, and compared among them (Figure 1a).

The MNCs were isolated from UCB. Before sorting, cryopreserved UCB cells were stained with 7-amino actinomycin D (7-AAD) live/dead dye and various blood cell markers. The population excluding debris from total UCB cells was 73.9% (R1 in Figure 1b), and the population existing as singlets was 72.03% of the total cells (R2 in Figure 1b). Among single cells, only living cells were gated using 7-AAD dye. The live cell population was 56.65% of the total cells. (R3 population in Figure 1b). Living cells were separated into CD34+ cells and CD34− cells by CD34 antibody, with CD34+ cells accounting for 0.2% of the total UCB (R4 population in Figure 1b) and CD34− cells accounting for 56.43% of the total UCB (R5 population in Figure 1b). The CD45 expressed cells were separated from CD34− cells and the ratio was 52.83% of total UCB (R6 in Figure 1b). In the CD34−CD45+ gating, the CD3−CD56+ population was sorted, and the remaining CD56+ cells were separated.

The cells remaining after sorting the CD56+ cells (CD56− cells) were 51.76% of the total UCB (R7+9 population in Figure 1b), and CD3−CD56+ cells were 0.87% of the total UCB (R10 population in Figure 1b). The CD34+ population consists of HSCs (UCB-HSCs), the CD3−CD56+ population consists of NK cells (UCB-NK cells), and the CD56− population consists of non-NK cells remaining after sorting out the CD56+ cells (UCB-nNK cells). The three sorted populations were used as origin cells. (Figure 1b).

The 34+, 56+, and 56− were generated iPSCs, respectively. They exhibited the typical cell morphology of human PSCs (Figure 1c). All iPSC lines were confirmed normal diploid through chromosomal copy number (Figure 1d) and pluripotency through teratoma formation (Figure 1e).

### 2.2. Comparison of NK Cells from Various iPSC Origins in Umbilical Cord Blood

**Figure 2:**
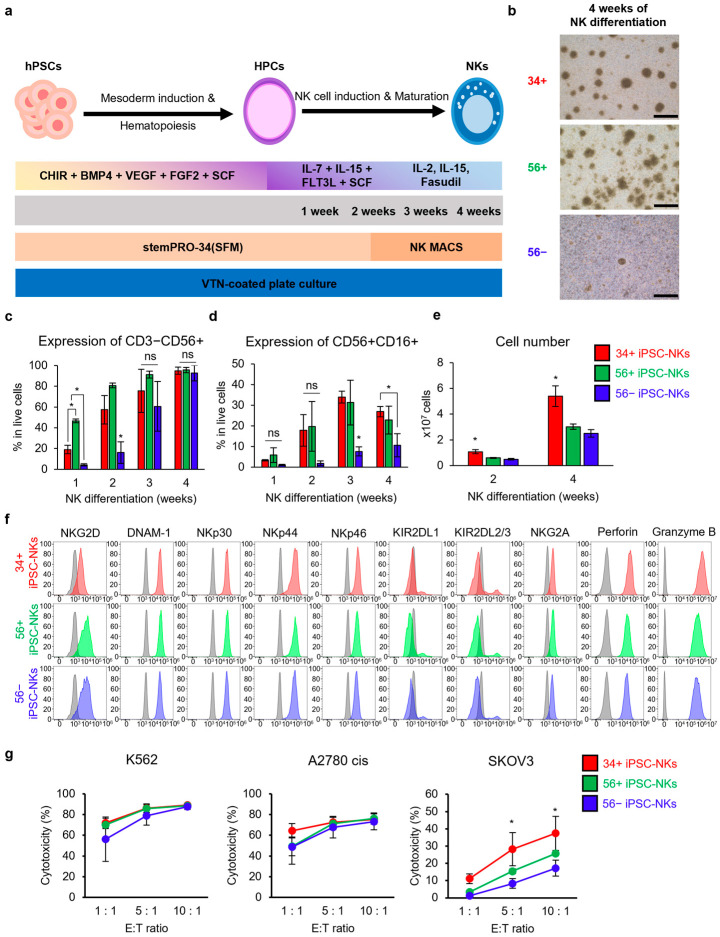
Differentiation of NK cells from 34+, 56+, and 56− hiPSCs. (**a**) Schematic representation of the differentiation protocol of NK cells from hPSCs using small molecules and cytokines without a feeder. (**b**) Morphology of hiPSC-NKs at 4 weeks of NK cell differentiation. The cultured cells were suspended and formed small colonies. Scale bar: 50 µm. (**c**) Expression of CD3−CD56+ in differentiated NK cells. The 34+ iPSC-NKs and 56+ iPSC-NKs showed significantly high expression at 1 and 2 weeks of NK cell differentiation. (**d**) Expression of CD56+CD16+ in differentiated NK cells. The 34+ iPSC-NKs and 56+ iPSC-NKs showed significantly high expression at 3 and 4 weeks of NK cell differentiation. (**e**) The cell growth of differentiated NK cells. The number of 34+ iPSC-NKs was significantly higher at both 2 and 4 weeks of NK cell differentiation. (**f**) Expression of NK cell surface receptors (activating receptors and inhibitory receptors) and cytolytic granules. The expression levels of all markers, excluding NKG2D, were similar in the three cell lines. The marker expressions of 34+ iPSC-NKs (Red), 56+ iPSC-NKs (Green), and 56− iPSC-NKs (Blue) were compared to those of the unstained cells (Grey). (**g**) Cytotoxicity assay against several cancer cell lines. The iPSC-NKs and cancer cells were used as effectors and target cells, respectively. Cytotoxicity against SKOV3 cells was significantly higher in 34+ iPSC-NKs than with 56+ iPSC-NKs and 56− iPSC-NKs at E:T = 5:1 and 10:1. Statistical analysis was performed using one-way ANOVA for (**c**–**e**,**g**) (*n* = 3). Data are shown as mean ± SD for (**c**–**e**,**g**). * *p* < 0.05. hPSCs: human pluripotent stem cells. HPCs: hematopoietic progenitor cells. NKs: natural killer cells. hiPS: human induced pluripotent stem cells. ns: not significant.

To investigate potential differences in the NK cell differentiation rate in the three types of iPSCs—34+ iPSCs, 56+ iPSCs, and 56− iPSCs—the iPSC lines were differentiated into NK cells (34+ iPSC-NKs, 56+ iPSC-NKs, and 56− iPSC-NKs), according to an established protocol (without feeder and EB formation) (Figure 2a). At 4 weeks of NK cell differentiation, all three types of iPSCs showed similar cell morphology and produced cell colonies (Figure 2b).

The 56+ iPSC-NKs showed a faster increase in CD3−CD56+ expression than other lines, followed by 34+ iPSC-NKs and 56− iPSC-NKs (Figure 2c, *p* < 0.05). However, all three types of iPSC-NKs exhibited similar purity, with a CD3−CD56+ ratio exceeding 90% at 4 weeks after differentiation from hematopoietic progenitor cells (HPCs) to NK cells (Figure 2c). The expression level of CD56+CD16+ was comparable in 34+ iPSC-NKs and 56+ iPSC-NKs, but was significantly lower in 56− iPSC-NKs at 3 and 4 weeks of NK cell differentiation (Figure 2d, *p* < 0.05). The 34+ iPSC-NKs displayed significantly more cell growth than the other two cell lines (Figure 2e, *p* < 0.05). The iPSC-NKs derived from three types of iPSCs were analyzed for the expression of cell surface markers, such as activating receptors (NKG2D, DNAM-1, NKp30, NKp44, and NKp46) and inhibitory receptors (KIR2DL1, KIR2DL2/3, and NKG2A), and cytolytic granules (perforin and granzyme B) (Figure 2f). All three types of iPSC-NKs showed similar expression of all surface markers (Figure 2f).

In cytotoxicity assays using a leukemia cell line (K562) and ovarian cancer cell lines (A2780 cis and SKOV3), all three types of iPSC-NKs exhibited no significant differences in cytotoxicity at each ratio against K562 and A2780 cis cells. However, the cytotoxicity of 34+ iPSC-NKs against SKOV3 cells at ratios of 5:1 (34+ iPSC-NKs: 28.2%, 56+ iPSC-NKs: 15.4%, and 56− iPSC-NKs: 8.3%) and 10:1 (34+ iPSC-NKs: 37.4%, 56+ iPSC-NKs: 25.6%, and 56− iPSC-NKs: 17.2%) was significantly higher than that with 56+ iPSC-NKs and 56− iPSC-NKs (Figure 2g, *p* < 0.05).

In summary, our data demonstrate the differentiation of three types of iPSCs into NK cells, with dramatic cell growth without feeder cells. iPSC-NKs exhibited a mature phenotype of NK cells. The 34+ iPSC-NKs demonstrated higher levels of cell number and cytotoxicity against the SKOV3 cell line than iPSC-NKs. Thus, 34+ iPSCs can produce more mature and functional NK cells than iPSCs from other origins.

### 2.3. Comparison of NK Cells from among ESC Lines

**Figure 3:**
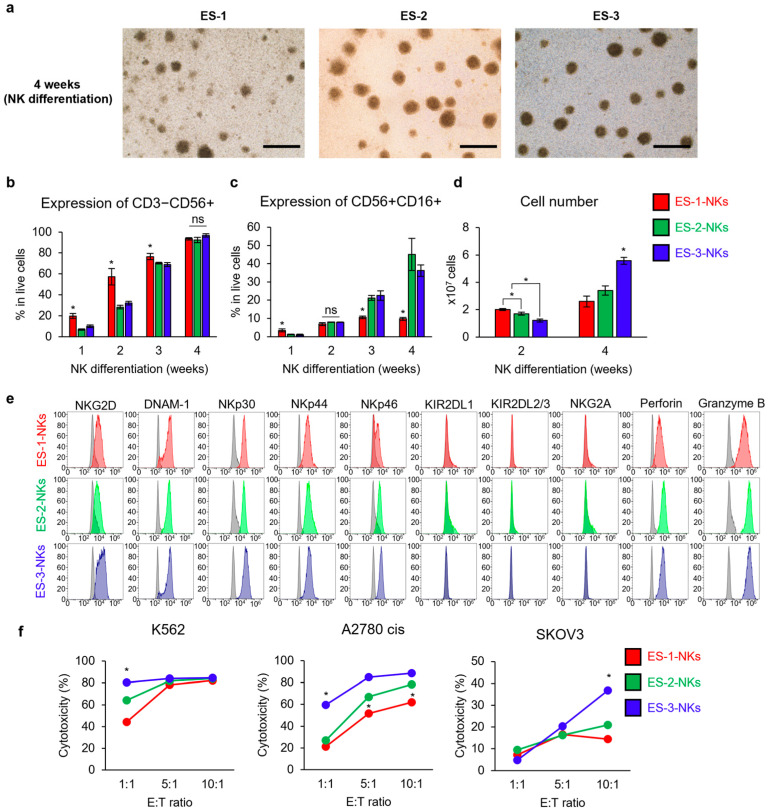
Differentiation of NK cells from various ESCs. (**a**) Morphology of hESC-NKs at 4 weeks of NK cell differentiation. The cells formed small colonies and were suspended. Scale bar: 50 µm. (**b**) Expression of CD3−CD56+ in differentiated NK cells. ES-1-NKs showed significantly higher expression at 1–3 weeks of NK cell differentiation. (**c**) Expression of CD56+CD16+ in differentiated NK cells. ES-2-NKs and ES-3-NKs showed significantly higher expression at 3 and 4 weeks of NK cell differentiation. (**d**) The cell growth of differentiated NK cells. The number of ES-3-NKs was significantly high at 4 weeks of NK cell differentiation. (**e**) Expression of NK cell surface receptors (activating receptors and inhibitory receptors) and cytolytic granules. No significant differences in expression levels were observed in NK cells derived from the three ESC cell lines. The marker expressions of ES-1-NKs (Red), ES-2-NKs (Green), and ES-3-NKs (Blue) were compared to those of their respective unstained cells (Grey). (**f**) Cytotoxicity assay against multiple cancer cell lines. The ESC-NKs and cancer cells were used as effectors and target cells, respectively. The ES-3-NKs showed significantly higher cytotoxicity than ES-1-NKs and ES-2-NKs in all three cancer cell lines. Statistical analysis was performed using one-way ANOVA for (**b**–**d**) (*n* = 3) and Fisher’s exact test for (**f**). Data are shown as mean ± SD for (**b**–**d**). * *p* < 0.05. ns: not significant.

We investigated differences in the NK cell differentiation rate among the three ESC cell lines (ES-1, ES-2, and ES-3), which were differentiated into HPCs for 2 weeks and then into NK cells (ES-1-NKs, ES-2-NKs, and ES-3-NKs) for 4 weeks (Figure 3a). These three ESC cell lines were characterized as previously described [31]. ES-1-NKs had significantly higher expression of CD3−CD56+ than ES-2-NKs and ES-3-NKs for up to 3 weeks after differentiation, but no significant difference was observed at 4 weeks (Figure 3b, *p* < 0.05). At 4 weeks, CD56+CD16+ expression was significantly lower in ES-1-NKs than in ES-2-NKs and ES-3 NKs (Figure 3c, *p* < 0.05). The cell growth was significantly higher in ES-3-NKs than in ES-1-NKs and ES-2-NKs at 4 weeks (Figure 3d, *p* < 0.05). However, no differences were found in the expression of activating receptors, inhibitory receptors, and cytolytic granules (Figure 3e).

Next, we performed a cytotoxicity assay to determine whether various ESCs showed functional differences in differentiated NK cells for each cell line. The cytotoxicity of ES-3-NKs was significantly higher than that of the other two cell lines when cocultured with K562 (ES-1-NKs: 44.12%, ES-2-NKs: 64.22%, and ES-3-NKs: 80.42%) or A2780 cis (ES-1-NKs: 21.2%, ES-2-NKs: 26.9%, and ES-3-NKs: 59.6%) at a 1:1 ratio and with SKOV3 at a 10:1 ratio (ES-1-NKs: 14.4%, ES-2-NKs: 20.9%, and ES-3-NKs: 36.8%) (Figure 3f, *p* < 0.05).

ES-1-NKs exhibited significantly lower cytotoxicity against A2780 cis at both 5:1 (ES-1-NKs: 51.7%, ES-2-NKs: 66.9%, and ES-3-NKs: 84.8%) and 10:1 (ES-1-NKs: 61.8%, ES-2-NKs: 78.3%, and ES-3-NKs: 88.5%) ratios than the other two cell lines (Figure 3f, *p* < 0.05).

In conclusion, each ES-NK cell line had a similar phenotype with a different differentiation pattern. However, some ES-NK lines could have better functions, such as ES-3.

### 2.4. Comparison of NK Cells from between ES3 and CD34+ iPSCs

We selected 34+ iPSCs and ES-3 from the three iPSC and three ESC lines, respectively, and compared their differentiation ability and function to determine which cell line is more effective in differentiating into NK cells and suitable for clinical applications.

**Figure 4:**
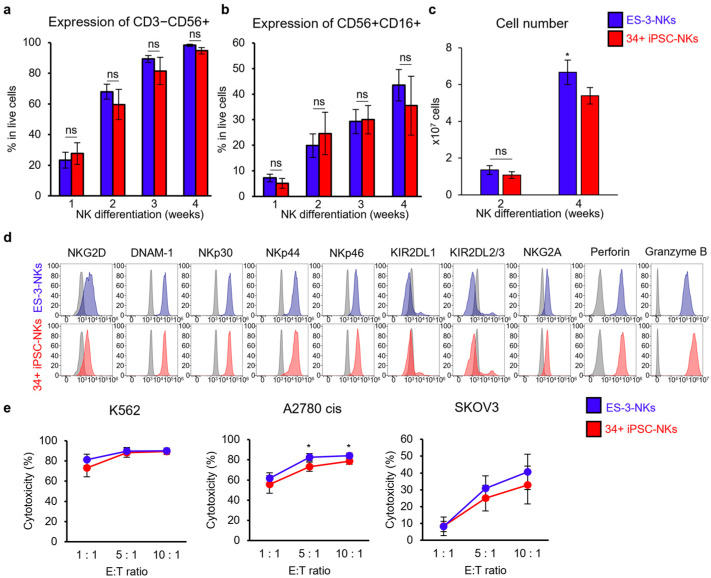
Comparison of NK cell differentiation between ES-3-NKs and 34+ iPSC-NKs. (**a**,**b**) Expression of CD3−CD56+ and CD56+CD16+ in differentiated NK cells. ES-3-NKs and 34+ iPSC-NKs showed similar expression during NK cell differentiation. (**c**) The cell growth of differentiated NK cells. The number of ES-3-NKs was significantly higher than 34+ iPSC-NKs at 4 weeks of NK cell differentiation. (**d**) Expression of NK cell surface receptors (activating receptors and inhibitory receptors) and cytolytic granules. No significant differences in expression levels were observed between ES-3-NKs and 34+ iPSC-NKs. The marker expressions of ES-3-NKs (Blue) and 34+ iPSC-NKs (Red) were compared to those of the unstained cells (Grey). The 34+ iPSC-NK data in this data are the same as the 34+ iPSC-NKs data in Figure 2f. (**e**) Cytotoxicity assay against multiple cancer cell lines. Differentiated NK and cancer cells were used as effectors and target cells, respectively. ES-3-NKs showed significantly higher toxicity than 34+ iPSC-NKs against A2780 cis cells at E:T ratios of 5:1 and 10:1. Statistical analysis was determined using Student’s *t*-test for (**a**–**c**,**e**) (*n* = 3). Data are shown as mean ± SD for (**a**–**c**,**e**). * *p* < 0.05. ns: not significant.

The expression levels of CD3−CD56+ and CD56+CD16+ between ES-3-NKs and 34+ iPSC-NKs during NK cell differentiation were not significantly different (Figure 4a,b).

ES-3-NKs showed different expression levels of CD3−CD56+ and CD56+CD16+ at the early stage of differentiation in individual experiments (Figure 3b,c and Figure 4a,b).

However, the expression levels were similar at the end of differentiation, suggesting that the early stage of NK cell differentiation can be variable even though the end stage of differentiation is consistent. The ES-3-NKs showed significantly higher cell growth than 34+ iPSC-NKs (Figure 4c, *p* < 0.05). A phenotype analysis between ES-3-NKs and 34+ iPSC-NKs revealed a similar expression pattern of various receptors and cytolytic granules (Figure 4d). However, the data for ES-3-NKs in Figure 3e, especially the expression levels of NKG2A, show differences, which could vary slightly with each differentiation.

In the cytotoxicity test, ES-3-NKs and 34+ iPSC-NKs exhibited similar cytotoxicity against K562 and SKOV3 cells (Figure 4e). However, ES-3-NKs demonstrated higher cytotoxicity than 34+ iPSC-NKs against A2780 cis cancer cells at ratios of 5:1 (ES-3-NKs: 82.6% and 34+ iPSC-NKs: 73.3%) and 10:1 (ES-3-NKs: 84.2% and 34+ iPSC-NKs: 78.7%) (Figure 4e, *p* < 0.05).

### 2.5. Comparison of NK Cells B2M Knockout and Wild ESCs

**Figure 5:**
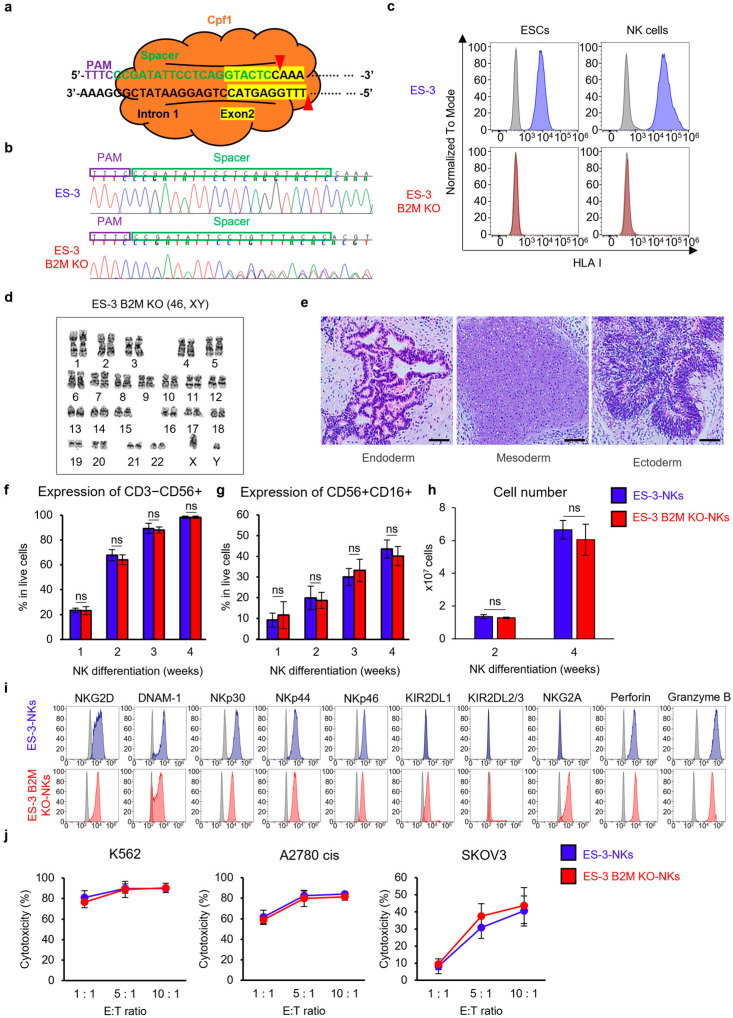
Characterization of ES-3 B2M KO cell line and comparison of NK differentiation between ES-3-NKs and ES-3 B2M KO-NKs. (**a**) Schematic image for knocking out the human B2M gene. Green indicates the target sequence of the human B2M gene. (**b**) Sanger sequencing results of ES-3 and ES-3 B2M KO. The targeted B2M sequence was knocked out. (**c**) HLA I (MHC I) expression in ES-3 and ES-3 B2M KO by flow cytometry. HLA I was not expressed not only in PSCs but also in differentiated NK cells. The marker expressions of ES-3-NKs (Blue) and ES-3 B2M KO-NKs (Red) were compared to those of their respective unstained cells (Grey). (**d**) G-banding karyotypes of ES-3 B2M KO cell line. ES-3 B2M KO showed a normal karyotype. (**e**) Formation of teratoma containing three germ layers (ectoderm, mesoderm, endoderm) by ES-3 B2M KO cell line. Scale bar: 200 μm. (**f**,**g**) Expression of CD3−CD56+ and CD56+CD16+ in differentiated NK cells. ES-3-NKs and ES-3 B2M KO-NKs showed comparable expression during NK cell differentiation. (**h**) The cell growth of differentiated NK cells. The number of differentiated NK cells was similar between ES-3-NKs and ES-3 B2M KO-NKs. (**i**) Expression of NK cell surface receptors (activating receptors and inhibitory receptors) and cytolytic granules. The marker expressions of ES-3-NKs (Blue) and ES-3 B2M KO-NKs (Red) were compared to those of their respective unstained cells (Grey). The ES-3 data in this data are the same as the ES-3 data in Figure 3e. (**j**) Cytotoxicity assay against multiple cancer cell lines. Cytotoxicity of ES-3-NKs and ES-3 B2M KO-NKs was similar against all three cancer cell lines. The ES-3 data in this data are the same as the ES-3 data in Figure 4e. Statistical analysis was determined by Student’s *t*-test for (**f**–**h**,**j**) (*n* = 3). Data are shown as mean ± SD for (**f**–**h**,**j**). ns: not significant.

To investigate whether B2M knockout (B2M KO) affects the differentiation of PSCs into NK cells and the function of differentiated NK cells, we knocked out the B2M gene in the ES-3 cell line (Figure 5a). In ES-3 in which B2M was knocked out (ES-3 B2M KO), it was confirmed through Sanger sequencing that the target sequence of B2M was knocked out (Figure 5b). The expression of MHC I was confirmed in PSCs and differentiated NK cells. As a result, it was confirmed that B2M was knocked out in the PSC state, and it was observed that MHC I knockout was maintained even after NK cell differentiation (Figure 5c). We performed karyotype analysis to determine whether B2M knockout affected the karyotype of PSCs. ES-3 B2M KO had all chromosomes without abnormal morphology (Figure 5d). Further, teratoma formation was also confirmed (Figure 5e), suggesting that pluripotency of ES-3 B2M KO was maintained after B2M knockout.

NK cell education has been studied over the past few years through the correlation between NK cell inhibitory receptors and MHC I [26,27,28,29]. Therefore, we compared the differentiation capacity between wild-type ES-3 and ES-3 B2M KO. There was no significant difference in CD3−CD56+ (Figure 5f) and CD56+CD16+ (Figure 5g) expression. Furthermore, ES-3-NKs and ES-3 B2M KO-derived NK cells (ES-3 B2M KO-NKs) showed similar cell growth (Figure 5h). A phenotype analysis between ES-3-NKs and ES-3 B2M KO-derived NK cells showed similar expression patterns, except for KIR2DL1 and NKG2A. ES-3 B2M KO-NKs displayed higher expressions of KIR2DL1 and NKG2A than ES-3-NKs (Figure 5i), which could be variable in individual differentiation. Although some receptors were differentially expressed between ES-3 B2M KO-NKs and ES-3-NKs, the cytotoxicity against all cancer cell lines (K562, A2780 cis, and SKOV3) was similar (Figure 5j). These data suggest that B2M knockout in PSCs does not affect differentiation into NK cells and the function of differentiated NK cells.

## 3. Discussion

We differentiated several types of hPSCs, including hiPSCs derived from CD34+, CD3−CD56+, and CD56− cells in UCB, three lines of hESCs (ES-1, ES-2, ES-3), and B2M-KO-ESCs, to NK cells and their capacities, such as cell growth and cytotoxicity, were analyzed. All hPSCs were successfully differentiated into NK cells, however, the efficacy varied among hPSC lines. The 34+ iPSC-NKs and ES-3-NKs demonstrated higher growth rates and cytotoxicity among hiPSC and hESC lines, respectively, and ES-3-NKs showed better efficacy than 34+ iPSC NKs. B2M-KO NK cells were similar to wild-type NK cells. These findings indicated that the screening for differentiation of PSCs into NK cells is required prior to selecting the PSC lines for the development of NK cell immunotherapy for universal allotransplantation.

NK cells are known to not cause GvHD in recipients, which occurs through HLA recognition by mediating T cell receptors (TCRs), and protect healthy tissues through KIRs and NKG2A signaling [32]. KIR mismatch can enhance the antitumor response [32]. Therefore, NK cells from healthy donors can be safely used in cancer treatment despite their HLA mismatch (mainly haploidentical allogeneic transplantation), allowing for off-the-shelf production [25,32].

Initial studies on NK cell-based ICT used primary NK cells, such as PBMC-derived NK cells and cord blood-derived NK cells. However, these primary cells have the disadvantage of requiring ex vivo proliferation with materials such as cytokines, because the amount that can be obtained from donors is limited [33].

One of the other sources of NK cells, the immortalized cell line NK-92, can overcome the disadvantage of primary cells through infinite proliferation. However, NK-92 cells exhibit aneuploidy and instability and require irradiation before ICT, which causes inhibition of proliferation in the patient [33].

PSCs can differentiate into all three germ layers, making them one of the sources of NK cells. They can be differentiated into various cell types for clinical application, including PSC-derived NK cells [22]. Because PSCs have high proliferative ability, large numbers can be obtained. Their high proliferation ability facilitates genetic modification compared with primary cells, which have low proliferation ability. These genetic modifications enable differentiation into NK cells, which exhibit strong anticancer effects through the expression of receptors such as CAR [34]. Despite these advantages, unclear molecular mechanisms and epigenetics influences during reprogramming or development lead to uncertainty about which PSCs are optimal for NK cell differentiation [35].

UCB-derived NK cells have been reported to have an immature phenotype, including low expression levels of CD16, KIRs, DNAM-1, NKG2C, IL-2R, and granzyme B [36]. However, the low expression level of KIRs could have an advantage for HLA-independent cytotoxicity during allogeneic transplantation [37]. Additionally, NK cells differentiated from UCB-derived iPSCs expressed high levels of KIRs and were more similar to PB-derived NK cells than UCB-derived NK cells [19]. These previous studies indicate that UCB-derived NK cells and NK cells differentiated from UCB-derived iPSCs can exhibit different phenotypes, suggesting that screening the phenotype of PSC-derived NK cells is essential for selecting PSCs for clinical applications.

Our data demonstrate that differentiating various PSCs into NK cells in vitro yields high-purity CD3−CD56+ NK cells within approximately 4 weeks after NK cell differentiation. Remarkably, even without feeder cells, we achieved substantial cell growth—1600–4400 times the number of initially seeded cells. All PSC-NKs exhibited a phenotype like that of mature NK cells, with no significant differences. Importantly, the cytotoxicity of these cells was high against the leukemia cell line K562 and ovarian cancer cell line A2780 cis and moderate against SKOV3 cells. These findings strongly suggest that our differentiated PSC-NKs can serve as effective effectors against target cells. In addition, we confirmed the expression of CD16 in NK cells differentiated from PSCs. This CD16 can recognize antibody-coated cancer cells and respond more strongly to them through ADCC [17,18]. However, it is necessary to verify the increase in cytotoxicity mediated by CD16 through ADCC and the degranulation capacity in our differentiated NK cells.

The differentiation rates of NK cells derived from different origins were similar. However, cell growth and cytotoxicity were different. Although our results demonstrated that ES-3 NKs had higher cell numbers and cytotoxicity compared to 34+ iPSC-NKs, our findings do not apply to all PSCs, as we used iPSCs derived from a single donor. Therefore, further studies should compare iPSCs derived from different donors. Additionally, our results showed varying expression levels in phenotype analysis for NK cells derived from the same PSCs, suggesting that individual experiments could exhibit slightly different results. Consequently, multiple replications are required before selecting PSCs for NK differentiation for clinical applications.

We suggest that it is important to screen PSC cell lines prior to genetic manipulation such as CAR. The PSC-derived NK cells genetically engineered through PSC cell line screening will have improved killing ability against specific cancer cells. To demonstrate this, we plan to insert the CAR gene into the PSCs to differentiate into NK cells and conduct research against various target cells.

Our results demonstrated that genetic manipulation, such as suppressing the expression of MHC I, in the PSC state does not significantly affect NK cell differentiation and their function. These findings may emerge as an evasion strategy against T cell-mediated rejection when PSC-derived NK cells are allotransplanted. However, the potential complications arising from MHC I knockout in the body remain unknown. Therefore, we recommend that in vivo experiments be conducted prior to clinical application.

## 4. Materials and Methods

### 4.1. UCB Cell Sorting

In total, 25 mL of frozen blood was thawed in a water bath for 5 min and diluted with DPBS (Welgene, Daegu, Republic of Korea, #LB 001-02) in a 1:1 ratio. Then, 10 mL of diluted blood was added to 3 mL of Ficoll (Cytiva, Amersham, UK, #17144002) and centrifuged at 900× *g* for 30 min. The buffy coat was collected with a pipette, and the sample was centrifuged at 700× *g* for 10 min. RBC lysis was conducted for 10 min. The cells were stained with 7-amino actinomycin D (7-AAD) Viability Staining Solution (Thermo Fisher, Waltham, MA, USA, #00-6993-50), and live or dead cells were analyzed on a flow cytometer (MoFlo, Beckman, Brea, CA, USA). Live cells were stained with CD34-PE (BD Pharmigen, Franklin Lakes, NJ, USA, #550761), CD45-V500 (BD Biosciences, Franklin Lakes, NJ, USA, #655873), CD3^−^FITC (eBioscience, Waltham, MA, USA, #11-0038-42), and CD56^−^APC (eBioscience #17-0567-42) antibodies and sorted into CD34+, CD3−CD56+, and CD56− cells (cells remaining after sorting out CD56+ cells). The data were analyzed using FlowJo version 10.8.1 software (Treestar Inc., Ashland, OG, USA).

### 4.2. Generation of iPSCs and PSCs Culture

UCB-derived CD34+ (UCB-HSCs), CD3−CD56+ (UCB-NK cells), and CD56− (UCB-nNK cells) cells were transduced to generate iPSCs with a CytoTune-iPS Sendai Reprogramming Kit (Thermo Fisher, #A16517) and characterized as previously described [31,38]. The CytoTune-iPS Sendai Reprogramming kit has been proven to show the absence of the Sendai virus in iPSCs across passages [39,40]. The generated iPSCs were characterized through CNV and teratoma assay as previously described [31].

The three ESC cell lines (ES-1, ES-2, and ES-3) were characterized previously [31]. The PSCs were cultured by the previously described, briefly, the cells were plated on a 60 mm cell culture dish coated with 5 µg/mL vitronectin in StemMACS medium at 37 °C in 5% CO_2_.

### 4.3. Copy Number Variation (CNV) Analysis

CNV analysis was performed using VeriSeq PGS Kit (Illumina, San Diego, CA, USA) and MiSeq Reagent Kit v3 (Illumina). The total CNV for all chromosomes was plotted to visualize the ploidy of iPSCs using BlueFuse Multi version 4.5 Software (Illumina).

### 4.4. Teratoma Formation

The animal protocols were approved by the CHA University Institutional Animal Care and Use Committee (IACUC). Teratoma formation experiments were performed by injecting hPSCs into the femoral region of 7-week-old NOD-SCID Gamma mice (NSG, Gembioscience) using a 1 mL syringe (Korea Vaccine Co., Seoul, Republic of Korea). A total of 4 weeks after cell injection, the mice with tumors were euthanized, and teratomas were isolated, sectioned, and histologically characterized using hematoxylin and eosin to identify the presence of representative tissues.

### 4.5. In Vitro NK Cell Differentiation

The differentiation of NK cells from iPSCs and ESCs was conducted according to the laboratory’s protocol. The PSC cell lines were used between passages 25 and 30 for differentiation. Briefly, PSCs were seeded in 6-well culture dishes coated with 5 μg/mL vitronectin in StemMACS with 10 μM fasudil (AdooQ, Irvine, CA, USA, #A10381) for 2 d. They were cultured for 15 d with differentiation basal medium StemPro-34 (Gibco, Waltham, MA, USA, #10639011) supplemented with 2.5% supplement, 200 μg/mL human transferrin (BioGems, Westlake Village, CA, USA, #10-366-1), 2 mM L-glutamine (Gibco, #21051-024), 1% penicillin–streptomycin (P/S, Simply, Taiwan, #CC502-0100), 0.5 mM ascorbic acid (Sigma, St. Louis, MO, USA, #A-8960), and 0.45 mM 1-thioglycerol (Sigma, #M6145). At day 0, the cells were treated with 5 μM CHIR99021 in StemPro medium. From days 2–4, the medium was supplemented with 50 ng/mL of human bone morphogenetic protein 4, 50 ng/mL of human vascular endothelial growth factor, and 100 ng/mL of fibroblast growth factor 2 (FGF2). Subsequently, 50 ng/mL FGF2, 50 ng/mL human vascular endothelial growth factor, 10 μM SB431542, and 1 μM of retinoic acid were added from days 4–5. From days 5–11, 50 ng/mL of stem cell factor (SCF) and 10 ng/mL of FGF2 were added to the medium. From days 11–15, the medium was supplemented with 50 ng/mL of SCF, 10 ng/mL of interleukin 3, and 1 mM of valproic acid. From days 5–15, 0.1% polyvinyl alcohol was added to the medium.

After day 15 of hematopoietic progenitor differentiation, HPCs were further differentiated into NK cells using 10 ng/mL of IL-15, 20 ng/mL of IL-7, 20 ng/mL of SCF, and 10 ng/mL of Flt3 ligand with StemPro medium for 2 weeks. Then, the medium was replaced with StemMACS medium supplemented with 10% FBS, 1% P/S, 10 ng/mL of IL-15, 500 IU of IL-2, and 10 μM of fasudil. Half-media changes were performed every 3 d. The cytokines and chemicals used are listed in Table 1.

### 4.6. Flow Cytometry

For staining surface markers, the suspended cells were harvested into a 1.5 mL tube, washed with 2% FACS buffer (PBS + 2% FBS), and stained using diluted antibodies at 4 °C for 30 min in the dark.

For intracellular staining, the cells were fixed and permeabilized using 4% paraformaldehyde (Bio solution) and 0.2% PBST (PBS + 0.2% Tween20) at RT for 30 min. After 30 min, the cells were washed with 2% FACS buffer, stained with diluted antibodies, and washed with 2% FACS buffer. The stained cells were analyzed using a CytoFLEX flow cytometer (Beckman Coulter, Brea, CA, USA). The data were analyzed using FlowJo version 10.6 software (Treestar Inc., Ashland, OG, USA). Antibody information is listed in Table 2.

### 4.7. Human Cancer Cell Lines

Three types of cancer cell lines, K562 (ATCC, Manassas, VA, USA), A2780 cis (ECACC), and SKOV3 (ATCC, Manassas, VA, USA), were kindly provided by Prof. H. J. An, Department of Pathology, CHA Bundang Medical center, CHA University (Sungnam, Republic of Korea).

Briefly, K562 and A2780 cis cells were cultured in RPMI 1640 medium supplemented with 10% FBS and 1% P/S. SKOV3 was cultured in McCoy’s 5A medium containing 10% FBS and 1% P/S. Media change was performed on alternate days. Subculture was performed once a week, and in the case of A2780 cis, 1 μM cisplatin was added to the medium only on the day of subculture. All cell lines were cultured at 37 °C and 5% CO_2_.

### 4.8. Cytotoxicity Assay

NK cell cytotoxicity against tumor cells was evaluated using a CellTrace Far Red Cell Proliferation Kit (Invitrogen, Waltham, MA, USA, #C34572)/7-AAD flow cytometry assay. PSC-NK cells were mixed with far red-stained target cells at various E:T ratios and incubated for 4 h (each E:T ratio per sample mixed in three independent wells among V bottom 96 wells). Incubated cells from three independent wells per sample were collected into a 1.5 mL tube. The cells were resuspended in FACS buffer (PBS + 2% FBS) containing 7-AAD. The percentage of target cell lysis was analyzed using CytoFLEX flow cytometry and FlowJo software. The percentage of specific lysis was calculated based on fluorescence measurements of the fluorescent dyes relative to cancer cells alone, using the following formula:

Specific Lysis (%) = far red- and 7-AAD-stained cancer cells with cocultured NK cells (%) − far red- and 7-AAD-stained cancer cells without cocultured NK cells.

### 4.9. Production of ES-3 B2M KO Cell Line

B2M KO was performed as previously described [41]. Briefly, the target sequence for human genomic B2M selected through the web software Benchling (https://benchling.com/ accessed on 10 January 2021) was synthesized by Macrogen (Seoul, Republic of Korea). The B2M-specific CRISPR-Cpf1 expression vector was constructed by cloning the synthesized and annealed oligomers (5’-agatCCGATATTCCTCAGGTACTC-3′ and 5′-aaaaGAGTACCTGAGGAATATCGG-3′) into the pY108 lentiviral vector. To produce lentivirus, HEK293T cells were transfected with B2M specific CRISPR-Cpf1 expression vector, psPAX2, and pMD2.G using Lipofectamine transfection reagent (Invitrogen, #18324-012). Transfection was performed in Opti-MEM medium (Gibco, #319850-070) for 5 h at 37 °C and 5% CO_2_ incubator. After 5 h, the medium of 293FT cells was replaced with fresh DMEM medium (Gibco, #11995-065) containing 10% FBS and cultured for 48 h at 37 °C and 5% CO_2_ incubator. The medium of 293FT cells cultured for 48 h was collected, and the supernatant was harvested through centrifugation. Lentivirus was separated from the harvested supernatant using a 0.45 µm filter and concentrated using a Lenti concentrator (Origene, Rockville, MD, USA, #TR30025) according to the manufacturer’s protocol. To produce a stable ES-3 B2M KO cell line, resuspended lentivirus in culture media were added to ES-3 and were incubated for 48 h in the culture medium. After 48 h, 2 µg/mL puromycin (Sigma, #P8833) was added to the fresh culture medium for selection and the medium was replaced. Knockout of the B2M gene was confirmed through flow cytometry and Sanger sequencing. Flow cytometry was performed using a CytoFLEX flow cytometer (Beckman Coulter, Brea, CA, USA) by staining selected cells with HLA-ABC-APC (BD Bioscience, #562006) antibody, and data were analyzed using FlowJo version 10.6 software (Treestar Inc., Ashland, OG, USA). Sanger sequencing of PCR products from selected cells was carried out by Macrogen (Seoul, Republic of Korea) and data were analyzed using Sequencher version 4.10.1 software (Gene Codes Corporation, Ann Arbor, MI, USA).

### 4.10. Karyotyping

Karyotyping was carried out on the ES-3 B2M KO cell line by standard G banding.

### 4.11. Statistical Analysis

Data are presented as mean ± standard deviation. Statistical significance was determined using a *t*-test or the Fisher exact test between two groups and one-way ANOVA among three groups. * *p* < 0.05, ** *p* < 0.01, *** *p* < 0.001, and **** *p* < 0.0001 were considered statistically significant. Statistical analysis was performed using GraphPad Prism version 6.01 (GraphPad, San Diego, CA, USA).

## 5. Conclusions

Depending on the origins of iPSCs or different ESC lines, the differentiation pattern of NK cells and cytotoxicity against cancer cells were displayed differently. Since B2M-KO did not affect the efficacy of differentiation into NK cells and the function of differentiated NK cells, screening for differentiation of PSCs into NK cells before selecting the PSC line for the development of NK cell immunotherapy is an essential process for universal allotransplantation including chimeric antigen receptor (CAR).

## Figures and Tables

**Table 1:** Cytokines and chemicals used for NK cell differentiation.

Cytokines and Chemicals	Company	Identifier
CHIR99021	MedChemExpress, Monmouth Junction, NJ, USA	HY-10182
Bone morphogenetic protein 4	Peprotech, Cranbury, NJ, USA	120-05
Vascular endothelial growth factor	Peprotech	100-20
Fibroblast growth factor 2	Peprotech	AF-100-18B
SB431542	SelleckChem, Houston, TX, USA	S1067
Retinoic acid	Sigma	R2625
Valproic acid	Supelco, St. Louis, MO, USA	PHR1061
Polyvinyl alcohol	Sigma	363146
Stem cell factor	Peprotech	300-07
Interleukin 2	Peprotech	200-02
Interleukin 3	Peprotech	200-03
Interleukin 7	Peprotech	200-07
Interleukin 15	Peprotech	200-15
FLT3-ligand	Peprotech	300-19
Fasudil HCl	AdooQ	A10381

**Table 2:** NK conjugated antibodies for phenotype analysis.

Antibody	Company	Identifier
Antibodies for surface marker		
Anti-CD3 APC	BioLegend, San Diego, CA, USA	Cat#317318
Anti-CD56 PE	BioLegend	Cat#362508
Anti-CD16 FITC	BioLegend	Cat#302028
Anti-NKG2D FITC	eBioscience	Cat#11-5878-42
Anti-NKp30 APC	BioLegend	Cat#325210
Anti-NKp44 PE	BioLegend	Cat#325108
Anti-NKp46 PE	BD Biosciences	Cat#557991
Anti-DNAM-1 PE	BioLegend	Cat#338305
Anti-NKG2A PE	Beckman Coulter	Cat#Z199
Anti-CD158a FITC	BD Biosciences	Cat#556062
Anti-CD158b PE	BD Biosciences	Cat#559785
Antibodies for intracellular		
Anti-Granzyme B PE	eBioscience	Cat#12-8898-82
Anti-Perforin PE	eBioscience	Cat#12-9392-82

## Data Availability

Data is contained within the article.
